# Psychological trauma and access to primary healthcare for people from refugee and asylum-seeker backgrounds: a mixed methods systematic review

**DOI:** 10.1186/s13033-020-00404-4

**Published:** 2020-09-11

**Authors:** Clemence Due, Erin Green, Anna Ziersch

**Affiliations:** 1 School of Psychology, North Terrace, The University of Adelaide, Adelaide, 5001 Australia; 2 The Southgate Institute for Health, Society and Equity, Flinders University, GPO Box 2100, Adelaide, 5001 Australia

**Keywords:** Refugees, Asylum seekers, Trauma, Healthcare access, Systematic review

## Abstract

**Background:**

Several reviews have found that psychological trauma affects access to health care services, including mental health care, in the general population. People from refugee and asylum seeker backgrounds are more likely to have a mental illness than the general population, and experience a broad range of barriers and facilitators to service access. However, to date there has been no comprehensive consideration of the potential effect of psychological trauma on access to primary health care within this population.

**Methods:**

This paper provides a mixed-methods systematic review of literature which included any consideration of the relationship between psychological trauma and access to primary health care. A systematic search of Medline, PsychInfo, Scopus, Web of Science, Embase, CINAHL and Cochrane Library was conducted. Study eligibility criteria were empirical, peer-reviewed studies that considered the relationship between psychological trauma and access to, or use of, primary healthcare in resettlement countries for refugees (including asylum seekers). Papers were required to be written in English and published between 1998 and August 2019. Quality was assessed using the Multi-Methods Appraisal Tool. The search identified a total of 14 eligible studies (11 quantitative and 3 qualitative) which had explored this relationship in refugee and asylum seeker populations.

**Results:**

Overall, synthesis of findings indicated variable results with respect to the impact of psychological trauma on service access. Specifically, the review found that while rates of psychological trauma were high. Key themes were that while general health care access was comparable or greater than the general population, rates of mental healthcare specifically were low. In addition, included papers identified a range of barriers to service access—particularly somatisation, stigma and healthcare provide knowledge about psychological trauma.

**Conclusions:**

While there is a critical need for more research in this area, the study points to several key recommendations including training of general practitioners in relation to psychological trauma, ensuring culturally responsive services, and the use of interpreters. Finally, due to the levels of somatisation found in some studies, ensuring general practitioners understand the somatic element of psychological trauma—particularly within some groups of people from refugee backgrounds—is important.

## Background

People from refugee and asylum seeker backgrounds resettled in high-income countries are more likely to suffer from a mental illness than the general community due to a range of pre and post migration factors including experiences of war, torture, family separation, forced migration and resettlement in unfamiliar environments [1, 2]. This includes psychological trauma—most commonly diagnosed as post-traumatic stress disorder (PTSD)—with research indicating that people from refugee backgrounds are approximately 10 times more likely to experience psychological trauma than the general population [1]. In this context access to primary health care—including mental healthcare—is particularly critical for refugees and asylum seekers given its key preventative role and the fact that primary care often functions as a gateway to specialised services [3, 4]. Indeed, research indicates that pathways to mental healthcare for people with refugee and asylum seeker backgrounds can be complex and are often influenced by both system structures and individual level help seeking preferences (e.g., for traditional healers) [4]. In terms of definitions, for the purposes of this paper, a broad approach was taken to primary healthcare, which included some mental healthcare services where it was clear that these were offered by frontline services [5].

Evidence concerning best practice in interventions designed for psychological trauma is mixed, and this is particularly true for interventions when working with adults and children from refugee or asylum seeker backgrounds (hereafter ‘refugees’ [defined as those who have had their claims to asylum assessed and approved] except when referring specifically to asylum seekers [defined as those who are currently displaced and seeking refugee status]) [2]. In general, best practice care is considered to be holistic, community-oriented and culturally appropriate, with a view to building on strengths within individuals and communities rather than adopting a deficit based approach to treatment [2]. Testimonial-based psychotherapy (e.g. narration of memories) or emotion-focussed therapies (e.g. emotion recognition and regulation) are generally considered to form an important part of any trauma informed intervention, and these forms of intervention require specialised skills and training [6–8]. Overall, while informal and community supports should ideally form part of holistic care, engagement with the mental health system—or with trauma trained primary care workers—is a critical part of working with psychological trauma [6].

A range of barriers and facilitators of primary health care access have been identified for refugees. These include language requirements, cost, health and health system literacy, stigma (especially for mental health), availability of specialist services, training for health practitioners working with refugees, and the cultural appropriateness of care [4, 9–12]. More broadly, there are several key models conceptualising factors associated with health care access, with definitions of access varying across disciplines and researchers [13, 14]. This review follows Penchansky [15] in arguing that access can be conceived as the relationship between those who seek to access healthcare and the healthcare resources available to them. While not drawing upon a model specifically, the paper also reflects Levesque and colleagues [14] in viewing domains of access existing at both a service level (such as availability, affordability and appropriateness) and individual level (such as health beliefs and ability find or access services). In line with this, the term ‘access is used in this paper to cover both the ability to access services in the first place as well as rates of subsequent utilisation [14]. However, an evidence gap exists in relation to whether psychological trauma has a specific impact on primary health care service access and utilization for this population.

Research with the general population typically supports the argument that psychological trauma can affect health service access, including through trauma-specific barriers [16], although findings are mixed. Much of this research has been conducted with veterans, where PTSD has been associated with increased service access and use for mental health, with mixed findings for physical health [17–19]. However, it should be noted that these results may not be generalisable to either the general population or refugee populations due to the availability of specialised trauma services, the assistance with cost (e.g., compensation) available to veterans in most countries, and the fact that veterans are not seeking services in a new country. A systematic review of health service use predictors in people with PTSD found most included studies identified increased service access and use for people with PTSD, although some studies found no relationship and others found an inverse relationship (specifically, PTSD severity was associated with decreased use) [19]. Overall, the review found consistent evidence for increased mental health service use in women, people with longer trauma histories, and specific PTSD diagnoses. None of the included studies focused on refugees. A review by Kantor and colleagues [16] focusing on barriers and facilitators to mental health service utilisation in trauma survivors found trauma-specific factors did affect service access, particularly concerns about re-experiencing traumatic events, which resulted in lower service use. Other trauma-specific barriers included concerns about stigma and psychological comorbidities (particularly depression). The only factor specific to refugees noted in this review was the use of interpreters, and the review did not comment on the link between psychological trauma and service use for refugees more broadly.

This paper therefore provides a systematic review of the literature that has explored the impact of psychological trauma on primary healthcare service access and use for refugees living in resettlement countries. Specifically, we aimed to collate and synthesise the available evidence concerning how psychological trauma affects utilisation of primary health care and to identify barriers and facilitators to improve provision of and access to primary healthcare for this population.

## Methods

This systematic review utilised the PRISMA guidelines for conducting systematic reviews to explore the research questions concerning psychological trauma and primary healthcare access.

### Inclusion criteria

The inclusion criteria were empirical, peer-reviewed studies that considered the relationship between psychological trauma and access to, or use of, primary healthcare in resettlement countries for refugees. Primary healthcare here was considered in its broadest form and included mental health services where it was clear that the papers reported on service access from at least some mental health frontline providers. This approach was taken for several reasons, including recognition of the differences in healthcare systems across countries [5], to ensure that all papers discussing primary healthcare in any form were included, and since primary healthcare services play a key preventative function including in relation to mental health. Studies which focussed only on emergency services, tertiary medical centres or other in-patient services were excluded. Where the level of mental healthcare was unclear, a broad approach was taken, and the paper was included in the review. Additionally, where papers reported on both mental health services which were primary healthcare services as well as specialist services, all findings reported in the paper were provided.

Papers were required to be written in English and published between 1998 and December 2019. Studies needed to include specific reference to psychological trauma, or provide disaggregated trauma data if composite mental health data was used or comorbidities were discussed. As per the DSM-5, trauma is defined here as the psychological outcome of exposure to traumatic events and does not include the traumatic events themselves [20]. While PTSD is the standard clinical diagnosis associated with psychological trauma, inclusion criteria for this systematic review did not specify PTSD diagnosis given debates in the literature about whether PTSD is the only, or most accurate, diagnosis for refugees [6]. Instead, to meet inclusion criteria, papers simply needed to reference psychological trauma in any form. Papers that spoke only of exposure to traumatic events, however, were excluded. Papers that focused on internally displaced persons or migrants were also excluded, as were studies that referred to “migrants” or “immigrants” without providing sufficient information as to arrival status. Studies in non-resettlement countries (states without United Nations High Commission for Refugees (UNHCR) resettlement programmes in operation as of 2017) were also excluded, given that the scope of the review was to understand the experiences of healthcare in countries where refugees or asylum seekers were intending to stay in the longer term.

### Search strategy

Medline, PsychInfo, Scopus, Web of Science, Embase, CINAHL and Cochrane Library were searched with the help of a research librarian (full search terms can be found in Table 1).

**Table 1:** Search terms

Population	Psychological trauma	Intervention	Service
Refugee*	Trauma	Access	“primary healthcare”
“asylum seeker*”	PTSD	Contact	“primary healthcare service*”
“humanitarian visa*”	“post-traumatic stress disorder”		Doctor
	“mental health”		GP
	“mental illness”		“health practitioner”
	Distress		“medical practitioner”
	Psychological*		Psychologist
			“mental health service”
			Counsell*

An example Medline syntax was as follows: ((primary healthcare* or primary health care* or general practice* or GPs or family medicine or health servic*) adj4 (access* or use* or using or utili?ation or contact*)).tw,kw. AND ("mental ill*" or "mental disorder*" or trauma* or "post traumatic stress disorder*" or PTSD or "metal health*" or distress* or psycholog* or anxiety* or depress*).tw,kw. AND (refuge* or asylum seeker* or asylum-seeker* exile* or immigra* or migrant* or humanitarian*).tw,kw.

The reference lists of articles included in full text review were also manually searched for additional relevant articles. Article searches were conducted in English only.

**Fig. 1:**
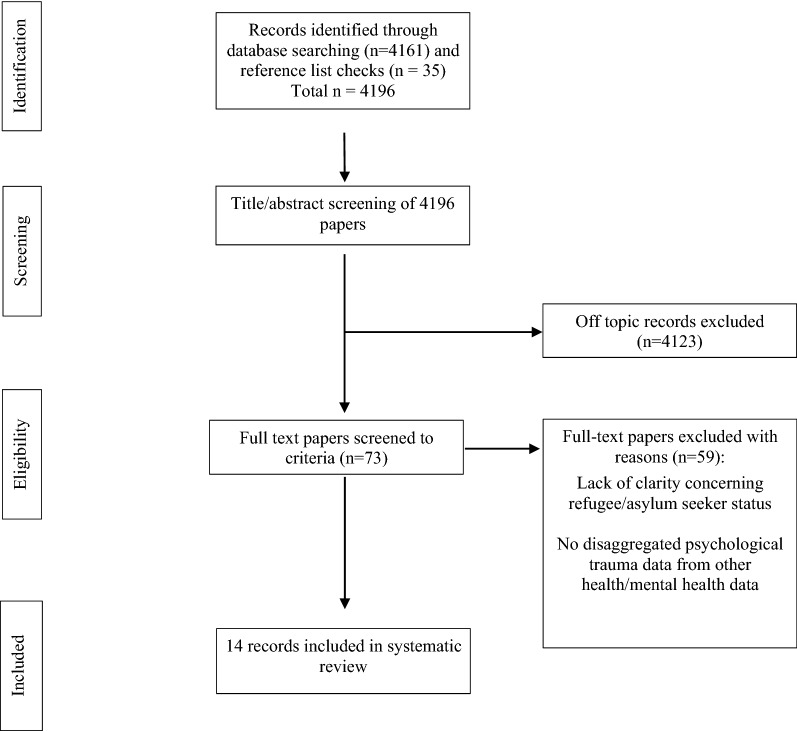
Search flowchart

The initial search returned 4161 results that were considered for inclusion, with a further 35 additional papers identified through the reference lists of included papers (see Fig. 1). All titles and abstracts and full texts were screened independently by the three authors, using Endnote software as outlined by Peters [21]. This resulted in a final sample of 14 independent studies.

### Data extraction and synthesis

Due to the diverse designs and aims of the studies, no meta-analysis was performed. Instead, results of the studies were synthesised using inductive thematic analysis, guided by Braun and Clarke’s approach [22, 23], with a specific focus on findings related to trauma and healthcare access. Specifically, all articles were read, with findings concerning psychological trauma and access to primary healthcare highlighted and then coded.

### Quality and bias

Given the diverse designs and since the quality of the articles found was taken to represent the state of the current literature (see [24]), article quality was not considered in relation to inclusion criteria. Discussions of quality and bias for the included papers are provided below in the results section.

## Results

From the initial 4196 results, 14 peer-reviewed papers met the inclusion criteria (see Fig. 1 above for search results).

### Description of studies

Specific details of each of the studies can be found in Table 2, while Table 3 provides an overall summary of study characteristics.

**Table 2:** Summary of included articles

Author/date	Aim/study focus	Study design	Sampling	Outcome focus/measures	Main findings relevant to PTSD and service access
Asgary et al. (2011)	To examine the experience of refugee asylum seekers accessing health care	Qualitative: focus groups and interviews	Purposive sampling from specific health clinic	Experiences of accessing healthcare	Asylum seekers did not make link between experiences of trauma and ongoing medical sequelae (or mental health) nor see psychological issues as appropriate for/requiring medical intervention
			Final sample of 50 (35 asylum seekers and 15 service providers)	Semi-structured interview schedule	Heavy burden of shame and stigma about sharing psychological trauma and histories of abuse
			85% male and mostly from African countries		Service providers identified mental illness and particularly experiences of trauma as a major obstacle to obtaining care
					Medical professionals may lack adequate training in recognizing the signs and symptoms of trauma
Bean et al. (2006)	To explore need for mental health care and patterns of utilization for unaccompanied minors	Quantitative: cross sectional survey	Purposive sampling from database for unaccompanied humanitarian minors	Patterns of service utilization	60% of the unaccompanied minors reported need for mental health care, but only 11.7% had received services
			Mixed sample (N = 3032): unaccompanied minors (n = 920), their legal guardians (n = 557), and their teachers (n = 496)	Stressful Life Events scale; Hopkins Symptom Checklist-37 (Adolescents); Reactions of Adolescents to Traumatic Stress; Child Behaviour Checklist; Teachers Report Form	Adolescent traumatic stress was a predictor for variables of ‘perceived need’ and ‘unmet need’, but not for ‘self-reported service use’
			Compared with a representative Dutch adolescent sample (n = 1059)		Number of stressful life events was the most crucial predictor for ‘perceived need’ and ‘unmet need’
Blair (2001)	To examine the extent and manifestation of mental health problems among Cambodian refugees in the USA, their rates of utilization of health and mental health services, and any barriers which may be preventing their access to these services	Quantitative: cross sectional survey	N = 124 randomly selected Cambodian refugees living in Utah	Mental health measured using the NIMH Diagnostic Interview Schedule (DIS)	Only 6% of participants had received medical care for PTSD
				PTSD measured using the Diagnostic Interview for Children and Adolescents (DICA-R)	Participants with a diagnosis of PTSD perceived a greater number of barriers that limited their ability to access health and /or mental health services compared to those without PTSD
				Questions concerning practical and cultural barriers to service use	
Colucci (2015)	To explore barriers and facilitators to mental health service delivery for refugee young people	Qualitative: focus groups and interviews	Purposive sampling. N = 115 service providers participated in focus groups plus five key informant interviews	Key facilitators and barriers to accessing and maintaining engagement with services	Using a trauma informed approach was identified as a key facilitator, while bring up trauma in mental healthcare too early was considered a barrier to ongoing maintenance of access
					Trauma was seen to impact on trust which can in turn impact service access
Geltman (2008)	To assess whether mental health counselling and other health services were associated with functional health outcomes of unaccompanied Sudanese refugee minors in the US	Quantitative: cross sectional survey	304 Sudanese refugee minors in foster care through the US Unaccompanied Refugee Minors Program (URMP)	Child Health Questionnaire	High prevalence of seeking care for somatic complaints (76%)
				Harvard Trauma Questionnaire	Those with PTSD were no more likely to have seen a mental health counsellor than those without PTSD, but were more likely to have seen *any* healthcare professional. However, authors note that treating patients with somatization may be challenging for those practitioners with limited experience
				Health services questionnaire developed by authors and based on questions adapted from the Health Care Access and Utilization section of the National Health Interview Survey (NHIS)	Lack of successful identification, diagnosis and treatment of the Sudanese refugees with worse functional and behavioural health, particularly PTSD
Jensen (2013)	To investigate how general practitioners experience providing care to refugees with mental health problems	Qualitative: interviews	15 service providers purposively sampled from areas with high proportions of migrants	Practitioners’ experiences of providing care	Participants felt that refugee patients with psychological trauma were too complicated for them to see on their own and were likely to refer to specialised services
			This paper focuses on 9 of the 15 interviews which were done with general practitioners specifically	Semi-structured interview schedule based around vignette	Only some participants expressed awareness of considering backgrounds of trauma when working with refugee clients
Lamkaddem (2014)	How to explain the persistently high prevalence of PTSD among resettled refugees despite the fact that various PTSD treatments are known to be effective?	Quantitative: longitudinal study design	Sampling unclear (described elsewhere)	Part IV of Harvard Trauma Questionnaire	21% of respondents with PTSD had contact with a mental health care provider at T1. This increased at T2 to 54%. However, at T1 those who reported using mental healthcare had greater PTSD symptom severity
			At T1: 410 refugees from Iran, Afghanistan and Somalia both with and without residence permits (178 asylum seekers and 232 residence permit holders)		In refugees who had used mental health care, PTSD symptoms generally improved
			At T2: 172 (all permit holders)		Low use of mental healthcare partly argued to explain steady PTSD rates across time in resettlement countries
Maier (2010)	To determine the current mental health status and patterns of healthcare utilisation	Quantitative: descriptive	Convenience sample from list supplied by Swiss Federal Office for Migration	Standardised Neuropsychiatric interview (MINI)	Asylum seeker participants incurred significantly higher healthcare costs than the comparable resident population and consult doctors more frequently but rarely receive specific treatment for their mental health problems
			78 adult asylum seekers	Records from Helsana health insurance company	Participants with a psychiatric disorder reported significantly more appointments than those without a psychiatric disorder
			After their first year of residence		
Sanchez-Cao (2013)	To describe the levels of psychological distress and mental health service contact among a group of unaccompanied asylum seeker children living in London	Quantitative: descriptive	Convenience sample	Harvard Trauma Questionnaire	High levels of psychological distress on self-report, with 66% at high risk of PTSD and 12% at high risk for depressive disorder. However, only 17% were in contact with mental health services, and this was predicted by depressive symptoms and time spent in the UK (not PTSD)
			71 unaccompanied minors	Impact of Event Scale	Depressive symptoms rather than post-traumatic symptoms best predicted service contact
				Birleson Depression Self-Rating Scale for children	
				Strengths and Difficulties Questionnaire self-report version	
				Attitudes to Health and Services Questionnaire	
Silove (2007)	To investigate the contribution of trauma and PTSD to the overall prevalence of mental disorders amongst Vietnamese refugees and the host Australian-born population	Quantitative: cross sectional with comparisons with Australian-born sample	Probabilistic sampling of Vietnamese refugees (n = 1161) resettled in Australia for 11 years (drawing on census data)	Composite International Diagnostic Interview (CIDI 2.0) with trauma events schedule expanded from the Harvard Trauma Questionnaire	In comparison to Australian non-refugees, Vietnamese refugees were more likely to focus on the somatic symptoms of PTSD
	To gauge patterns of service utilization for PTSD across the two populations		Australian-born sample (n = 7961) drawn from ABS Survey of Mental Health and Wellbeing		Australians and Vietnamese refugees with PTSD had high rates of general health consultations overall and specifically with primary care physicians. However, Australians with PTSD were almost twice as likely as Vietnamese refugees to indicate that these primary care consultations were for a mental health problem
					Approximately one in 10 Vietnamese refugees compared to one in three Australians with PTSD had consulted a specialist mental health professional
Slewa-Younan (2015)	To examine levels of psychological distress and help-seeking behaviour in resettled refugees attending English tuition classes in Australia, and their associations with participants demographic characteristics	Quantitative: cross sectional survey	N = 225 Iraqi refugees resettled in Western Sydney	Kessler Psychological Distress Scale (K-10)	Participants had high levels of distress and PTSD symptomatology and low uptake of mental health care
				Harvard Trauma Questionnaire	Of those with probable PTSD, the most common type of help sought was from a family member (23.1%), followed by GP (21.5%), psychiatrist (13.8%), psychologist (12.3%) and religious leader (10.8%)
Weine (2000)	To profile trauma related psychiatric symptoms in a group of refugees not seeking mental health services and to consider the service implications	Quantitative: descriptive	Two groups of Bosnian refugees:	Development of an 18 item Trauma Exposure questionnaire	Participants who had accessed services had higher PTSD symptom severity
			1) Those who had not presented for mental health services, recruited from Bosnian Refugee Center and two other contacts (N = 40)	PTSD Symptoms Scale	Participants who has not sought services also had substantial trauma-related symptom levels: of 40 participants not presenting for services, 28 (70%) met symptom criteria for PTSD diagnosis
			2) 29 Bosnian clients who had received services, access through a clinic	Center for Epidemiological Studies Depression Scale (CES-D)	
				MOS 36-Item Short-Form (SF-36)	
Wong (2015)	To examine US-based Cambodian refugees’ utilization of mental health services across provider types, levels of minimally adequate care, and mode of communication with providers	Quantitative: descriptive	Probabilistic sampling (further details not provided)	Composite International Diagnostic Interview v2.1 (CIDI)	More than half of the participants who met criteria for PTSD or major depression or both (52%, n = 127) obtained mental health services from at least one provider in the past 12 months, however this was typically for medication
			227 Cambodian refugees who met the past 12 month criteria for posttraumatic stress disorder (PTSD) or major depressive disorder or both		
Wright (2016)	To examine changes in institutional resource needs and utilization over 2 years in a newly arrived refugee sample, and to investigate the role of institutional resource need and utilization on mental health in the post-arrival period	Quantitative: longitudinal	298 adult Iraqi refugees randomly selected	Harvard Trauma Questionnaire	Participants reported a significant increase in PTSD symptoms between the 1 and 2 year interviews
		Refugees were interviewed three times. Baseline very soon after arrival, at 1 year and at 2 years		PTSD Checklist (PCL)—civilian version	Higher utilization of psychological services predicted an increase in PTSD symptoms
				Hospital Anxiety and Depression Scale (HADS)	
				A 14-item checklist was developed for assessing refugees needs for and utilization of institutional resources

**Table 3:** Overall summary of study characteristics

	Quantitative (N = 11)	Qualitative (N = 3)	Total N = 14
Year of Publication			
1998–2001	2		2
2002–2006	1		1
2007–2011	3	1	4
2012–2018	5	2	7
Region of study			
Australia/New Zealand	2	1	3
United Kingdom	1		1
United States	5	1	6
Netherlands	2		2
Switzerland	1		1
Denmark		1	1
Informant group			
Refugees/asylum seekers	10		10
Service providers		2	2
Refugees/asylum seekers and service providers	1	1	2
Sample size			
Under 50		2	2
51–100	3		3
101–200	1	1	2
201–300	3		3
301–500	2		2
500 +	2		2
Outcome focus			
Mental health care	5		5
General primary health care		2	2
Both	6	1	7

All 14 of the studies were peer reviewed papers: 11 quantitative [25–35] and three qualitative [25, 36, 37]. Eight studies included participation from refugees [25, 26, 30–35], three from asylum seekers [28, 29, 36], and one included both refugees and asylum seekers [27]. Two studies [34, 36] sampled both refugees/asylum seekers and service providers, while two studies [10, 37] involved service providers only. Three studies focussed on unaccompanied minors [26, 29, 34].

With respect to resettlement countries, all studies were conducted in nominally “Western” countries, with the largest number in the United States or Canada (N = 6).

Four studies included refugees and asylum seekers from a mixed range of ethnicities and countries of origin [28, 29, 34, 36]. The remaining eight papers included samples of refugees or asylum seekers from one or more ethnicity or nationality, specifically Cambodian [25, 32], Sudanese [26], Afghani, Iranian and Somalian [27], Vietnamese [30], Iraqi [31, 33], and Bosnian [35] participants.

Five studies explored access or utilisation of general primary health care [30, 31, 33, 36, 37], which may or may not have included mental health care, six focussed exclusively on mental health [10, 27, 29, 32, 34, 35], and three specifically mentioned a focus on both mental health and general primary healthcare [25, 26, 28].

### Quality of evidence base in the reported papers

Issues of quality in the reported, published papers, and consistent with systematic review protocols, were considered with reference to the Mixed Methods Appraisal Tool (MMAT) [39]. The MMAT allows an appraisal of quality on the basis of the following criteria: clarity of the research questions, whether the data allows consideration of research questions, and then—depending on the study methodology—questions related to sampling, measurements, and data analysis. Consistent with the MMAT, this section provides a broad overview of quality for all included articles according to the tool domains. Article quality was only considered with respect to the papers as they appeared in their published form. Authors were not contacted for further information about their studies given that the review explored a topic—psychological trauma and primary healthcare access—which was often different to the aims of the paper. It is recognised that some of the issues reported below may related to journal restrictions such as word counts or particular reporting requirements.

All studies had clearly articulated aims, satisfying the first criterion of the MMAT.

In relation to the qualitative studies, in all cases the findings were clearly derived from the data, the results were clearly based on the data, and there was a coherence between the data, the analysis and the conclusions. However, in the case of two studies [10, 36], interview questions were not provided in the published paper—although both provided exemplar extracts.

In relation to quantitative studies, all had collected data reported in the papers which allowed for the stated research questions to be answered. In two cases the sampling strategy was unclear, but details were provided in a paper published elsewhere [25, 26]. In all papers the sample was representative of the target population. Finally, while analyses were sometimes primarily descriptive they were all suitable to answering the research questions as stated in the papers.

In terms of potential sources of bias, thirteen of the fourteen included studies (quantitative and qualitative) used samples of convenience, with some samples recruited directly through mental health services, leading to potential sources of bias. The only study that used more robust sampling was Bean et al. 2006, who recruited minors through a register. Finally, studies which reported funding noted no conflict of interest, with funding sources largely philanthropic funds. Two potential exceptions to this are Maier et al. [28], who received funding from the Swiss Federal Office for Migration (which also helped to recruit participants), and Sanchez-Cao et al. [29], who received funding from the Westminster City Council Department of Social Services.

While not an issue of quality in relation to the published papers reported here, in relation to the aims of this review the analysis in some papers made consideration of the relationship between psychological trauma and access to services difficult to determine. For example, some analyses did not provide significance testing or effect sizes for the relationship between variables relevant to this review.

### Definitions, measures and instruments

A broad range of trauma measures were used. The Harvard Trauma Questionnaire (HTQ) was the most commonly used with five of the eleven quantitative studies including it in their data collection [26, 27, 29–31] followed by the Composite International Diagnostic Interview (CIDI-WHO) used by two studies [30, 32]. Other measures included the Stressful Life Events checklist [34], a version of the Hopkins Symptom Checklist [34], the Reactions of Adolescents to Traumatic Stress [34], the Child Behaviour Checklist [34], the Diagnostic Interview for Children and Adolescents [25], and the MINI [28], PTSD symptoms scale [35], and the PTSD Checklist [33]. All quantitative studies discussed the use of interpreters or translation/back-translation of measures.

In relation to ‘measures’ (e.g., interview questions), two [36, 37] did not specify interview questions with only one of the three [10] outlining an interview schedule.

### Prevalence of psychological trauma

Table 4 provides an overview of the prevalence rates for psychological trauma found within the studies where prevalence was measured (all of the quantitative studies except that by [34]). The study by Wong et al. [32] had specifically sampled for Cambodian refugees who met criteria for PTSD cut-offs in the previous 12 months. As such, their sample had prevalence of 97%, presumably due to the fact that 3% no longer met criteria after first measurement. All studies referred to psychological trauma as PTSD.

**Table 4:** PTSD prevalence/risk by study

First author (date)	Population ethnicity	Sample size	PTSD risk/prevalence (%)
Blair (2001)	Cambodian	124	45
Geltman et al. (2008)	Sudanese (minors)	304	20
Lamkadden et al. (2014)	Afgani, Iranian and Somalian	410	16
Maier et al. (2010)	Mixed	78	24
Sanchez-Cao et al. (2012)	Mixed (minors)	71	66
Silove et al. (2007)	Vietnamese	1161	4
Slewa-Younan et al. (2015)	Iraqi	225	40
Weine et al. (2000)	Bosnian	28	70
Wong et al. (2015)	Cambodian	227	97
Wright et al. (2016)	Iraqi	298	5

### Emergent themes

The thematic synthesis of the findings in the papers revealed mixed results regarding rates of access to primary health care (high rates of general health care access amongst those with PTSD and low rates of mental health care access), and a range of pathways by which trauma might affect health care access including somatisation, stigma, service provider knowledge and culturally appropriate services.

#### Rates of primary healthcare access

The review found that *general* primary healthcare access was typically high amongst those with PTSD (and typically higher than comparator groups of either refugees with low trauma symptomatology or non-refugee groups), however access to mental healthcare specifically was low.

#### General primary healthcare access

Three studies considered both mental and general healthcare access across PTSD and non-PTSD refugee groups [26], non-refugee comparator groups [30] or both [28]. All of these studies found higher service access for general or physical health in the PTSD group, although there was no difference in mental healthcare access specifically. For example, Geltman et al. [26] found that Sudanese refugee youth living in the United States who met criteria for a PTSD diagnosis (determined using the HTQ) were over twice as likely to have seen some type of healthcare practitioner than those who did not meet PTSD criteria. However, they were no more likely to have seen a mental healthcare practitioner in a primary healthcare setting. This was particularly the case for those youth with somatic complaints who were three times as likely to have seen a healthcare practitioner (but not necessarily one trained in mental health) and twice as likely to have sought emergency care. Similarly, based on health care records of refugees and the general population provided by an insurance agency in Switzerland, Maier et al. [28] reported rates of service access per year for their refugee participants with PTSD (N = 19 with an average of 18.7 appointments), any diagnosis (N = 32 with an average of 15.6 appointments) and no diagnosis (N = 46 with an average of 7.4 appointments). While there was no reported analysis of those with PTSD specifically in terms of comparisons (that is, only the raw data reported above was provided), a reported t-test indicated that refugees with a psychiatric disorder of any form had significantly more appointments per year than refugees without. Finally, in a comparative study between Australian-born participants and refugees from Vietnam, Silove et al. [30] found higher rates of general health consultations in those with PTSD in both groups of participants (e.g., 88.8% of Vietnamese participants with PTSD had accessed general services compared to 76.6% of those without; 91.3% of Australians with PTSD had accessed general health services compared to 86.1% of those without). Members of the general community with PTSD were almost twice as likely to indicate that their consultations were for mental health issues as compared to the Vietnamese refugees.

#### Mental healthcare access

Six studies focused solely on mental health access for refugee populations, four of which were cross sectional [29, 31, 34, 35] and two longitudinal [27, 33]. The four cross sectional studies found that overall rates of mental healthcare access were low compared to either population-level access in the relevant country or as would be expected based on the prevalence rates of PTSD found in the studies. Slewa-Younan et al. [31] explored the predictors of help seeking in a sample of 225 Iraqi refugees in Australia. Nineteen percent of the participants reported ever having sought help, including through primary healthcare, for a mental health problem. A significant association was found between PTSD symptomatology (measured by the HTQ) and help seeking behaviour in participants with PTSD. Specifically, those who met the threshold for clinically significant symptoms were two and a half times more likely to have sought help for a mental health problem that those below the threshold. However, the authors note that only 32.9% of the sample of those experiencing PTSD symptomatology said they had sought help for a mental health problem at all. The authors reported that the most common source of help seeking was family (23.1%) followed by a GP (21.5%) and then psychiatrists and psychologists at 13.8 and 12.3% respectively (raw numbers not provided). Only 9.2% of the participants had sought help from specialist torture and trauma services. Weine et al. [35] conducted a study with 70 Bosnian refugees in the US, exploring subgroups of 29 participants who had presented to services with 41 who had not. They found that all of the 29 participants who had accessed mental health services met PTSD cut off scores as measured by the PTSD symptom scale. 70% (N = 28) of the 41 participants who had not accessed mental health services also met symptom criteria for PTSD—a percentage which the authors note indicates gaps in mental health care for refugees with psychological trauma.

Bean et al. [34], in their study of unaccompanied minors from a range of countries currently living in the Netherlands, also found that psychological trauma (as measured by the Reactions of Adolescents to Traumatic Stress measure—prevalence rates not reported) positively predicted both perceived *need* to access mental health services and *unmet need*, measured through a survey instrument. However, a logistic regression model exploring predictors of service use that included reactions to traumatic stress was not statistically significant. On the other hand, Sanchez-Cao et al. [29] found that use of mental health services for unaccompanied minors did not differ between those who had PTSD (as measured by the HTQ) and those who did not. Overall service access was low (17%) and this was predicted instead by depression and time in the UK rather than PTSD.

In a longitudinal study, Lamkaddem et al. [27] conducted research across two time points (T1 and T2 seven years later) with refugees and asylum seekers from Iran, Afghanistan and Somalia living in the Netherlands. They found low rates of mental health care use at T1 for those with PTSD (21.4% had accessed care), with this increasing to 53.8% at T2. Mental healthcare at T1 was related to higher PTSD severity, but this analysis was not conducted for T2. The reported rates of access for this group were lower: 6% at T1 and 13% at T2. Mental healthcare use at T1 was significantly associated with improvement in PTSD scores between the two waves, although the authors noted that confidence intervals were large. Wright et al. [33] conducted a longitudinal study of the first two years of resettlement for Iraqi refugees living in the US (N = 298). Contrary to the hypotheses the study found that refugee participants reported a significant increase in PTSD between 1 and 2 year interviews, and that higher utilization of psychological services in the first two years predicted a significant increase in PTSD symptoms, which the authors argue could be due to the fact that people with declining mental health are more likely to seek help. Overall, both Lamkeddam et al. [27] and Wright et al. [33] found that service use was associated with higher PTSD rates, but findings differed in relation to the impact of service access with Lamkeddam et al. [27] finding improvement in PTSD while Wright et al. [33] found an increase in symptoms.

While the above studies all show a similar pattern of low service access, the study by Wong et al. [32] of US-based Cambodian refugees from a larger study specifically for PTSD diagnosis found that 52% of their sample of 227 participants had accessed mental health services in the past 12 months, with most seeing a psychiatrist (39%) followed by a general medical doctor (29%). In their discussion, the authors note that Cambodian refugees were accessing psychiatrists at almost double the rate of the general US population. Interestingly, the primary type of access appeared to be for medication rather than psychotherapy, which the authors note could be problematic due to concerns about prescriptions of psychopharmacological medications and the fact that best practice treatment includes trauma informed psychotherapy. Conversely, only four percent of participants reported seeing a “non-physician mental health professional” (presumably a psychologist or counsellor although this is not clear in the paper), compared to 19% of white Americans and 14% of Asian Americans.

### Pathways between trauma and service access: barriers and facilitators

A further theme identified in the included papers related a range barriers and facilitators to primary healthcare access which may be directly related to, or affected by, psychological trauma. This included general barriers, somatisation, stigma, service provider knowledge and culturally appropriate services, and ‘other barriers’.

### Somatisation

Of particular interest in relation to trauma is that two of the quantitative studies [26, 30] and two of the qualitative studies [36, 37] directly identified somatisation (e.g., experiencing psychological distress as somatic symptoms—[38]) as a key factor for the increase in utilization of general primary healthcare as compared to mental healthcare. Geltman et al. [26] noted in their discussion that the Sudanese minors in their study reported high levels of medical care for problems consistent with somatisation, with those patterns most common among those with PTSD, and that this presents a challenge for practitioners who may have limited experience with trauma. Similarly, Silove et al. [30] noted that somatisation of symptoms in their Vietnamese refugee population could explain the lower mental health service group in that population as compared to their Australian-born comparison group, and that there is a “cultural tendency” (p. 475) to somatise distress within Vietnamese people. In two qualitative studies [36, 37] service provider interviewees noted high levels of somatisation amongst refugees and asylum seekers experiencing psychological trauma. In these studies, service provider participants noted that somatisation may be associated with trauma symptomatology and may increase primary healthcare access since help seeking is then associated with physical, rather than psychological, complaints.

### Stigma

In their qualitative study of 35 asylum seekers and 15 service providers in the United States, Asgary and Segar [36] reported that interviewed asylum seeker and refugee participants were “resigned” (p. 509) to poor mental health, and service providers reported that shame and stigma associated with mental illness prevented help seeking, thereby acting as barriers to service access. Other studies also noted that stigma associated with mental health issues—particularly trauma—likely acted as a barrier to service access [33].

### Service provider experience and culturally appropriate services

All three of the qualitative studies found that psychological trauma acted as a barrier to service access (or retention within services) because service providers did not know how to work with refugees who were experiencing psychological trauma. In an Australian study of 115 service providers working within mental health settings, Colucci et al. [10] found that their service providers felt bringing up trauma-related issues too early in their sessions led to potential disengagement with services by their clients. Similarly, Jensen et al. [37] in their qualitative study of 15 general practitioners working with refugee clients in Denmark, found general practitioners often reported that psychological trauma was too complicated for them to work with. As such, participants in this study noted that they needed to refer clients to specialised services. Finally, Asgary and Segar [36], Colucci et al. [10], and Jensen et al. [37] all note the importance of trauma informed approaches in providing services to refugees experiencing psychological trauma.

### Other barriers

In their study of 124 Cambodian refugees in the US, Blair [25], found that those with PTSD identified more barriers than those without: an average of 5.1 barriers as compared to 3.5 for those without PTSD. For the sample overall (those with and without PTSD—this was not presented separately), the most commonly reported barrier was “I think American medical people do not understand Cambodian health problems” (n = 53; 43% of the total sample) followed by gaining better help from family (n = 51; 41%), not understanding required paperwork (n = 40; 32%), and language and literacy issues (n = 40; 32%).

Five other studies [27, 29–31, 33] provided some reflections on the potential pathways through which trauma might impact service access, although this was not included specifically in their analysis. Key pathways noted included lack of language fluency [27, 29, 30], health system literacy (e.g., limited awareness of available services; [27, 29, 31], lack of culturally appropriate services [27, 30], high mobility [29], and difficulty registering or being referred to services [29].

## Discussion

The studies included in this review reported variable findings with respect to the impact of psychological trauma on access to primary healthcare services. Overall the rates of psychological trauma were high, though there was significant variation, likely related to variable methodologies used in the studies including differences in sampling methods and sample characteristics as well as the trauma measures used. In general, most studies found that rates of access to mental health services were low while general healthcare access was comparable or greater than comparator groups (e.g. refugees without psychological trauma or the general population) [26, 28–31, 34]. This varies from previous research with other populations, such as that outlined in the review by Elhai and colleagues [19], which found that mental health access for people with PTSD was associated with higher service access and use, but supports other findings concerning the existence of trauma-specific barriers to accessing healthcare [16]. The current study also identified some key pathways through which trauma may influence service access for refugees, particularly in relation to somatisation which appears to influence access to services through leading people to access general or physical health services rather than those for mental health. In terms of mental health access, the findings of this study point to some key recommendations for service providers to ensure that refugee and asylum seeker populations are able to access services if required.

The papers included in this review reported rates of psychological trauma from 4 to 70% (excluding Wong et al. [32], who sampled for PTSD), reflecting previous research which has identified a wide range in reported prevalence rates for trauma in refugee populations [6, 40]. Whilst prevalence rates were not the main focus of this review, they warrant discussion here since the wide divergence in rates illustrates some of the complexities with research in this area, and the importance of accurate measurement of psychological trauma to facilitate health service access. Previous literature has suggested cultural differences in expressions of emotion and psychological distress as well as the specific traumatic experiences, which could help explain this discrepancy in rates, together with differences in the measures used [1, 29, 41–45]. There is therefore a pressing need for research to focus on both what constitutes trauma refugees in the first place, and how to measure the resulting construct [46, 47].

In terms of primary healthcare access, quantitative studies included in this review found that service use for refugees experiencing psychological trauma was generally higher than the comparative groups of either refugees without PTSD [26], the general population [30] or both [28]. This was the case across youth [26] and adults [28, 30]. It is possible, then, that PTSD symptomatology may lead refugees to access primary healthcare (but not mental health specific) services despite the barriers identified previously in other literature (e.g., transport, language, understandings of health systems, as well as somatisation [4, 13–15]).

While rates of general healthcare access appear to be higher for refugee groups with PTSD, this was typically not the case for mental health specific services, where access remained low. Importantly, these findings differ from much of the previous quantitative research concerning the relationship between trauma and health service use which has found that PTSD symptoms and severity predict higher service access and use [19], but do support other literature which has found no relationship for trauma-specific symptoms [16, 48].

Three studies found contrary findings to those outlined above [29, 32, 35] that may be explained by methodological considerations. Specifically, Weine [35] recruited participants through different methods for the two groups (e.g. those who had not accessed services using network analysis and those who had were directly recruited through a clinic), resulting in potential discrepancies in other demographic criteria, although this was not noted. Sanchez-Cao et al.’s [29] findings with minors living with foster carers echoes previous research with youth in care and issues relying on identification of symptoms by carers [48]. Finally, Wong et al.’s [32] sample was sourced specifically for a PTSD diagnosis and the high rates of mental health access they found was typically for medication rather than any other mental health care. Overall, this variability in methodologies and samples represented the heterogeneity of all included studies—an issue affecting much of the literature concerning trauma and service use—which makes drawing conclusions difficult [19]

The included papers identified a range of barriers and facilitators to accessing mental health care, including some trauma-specific factors. These included barriers such as stigma associated with psychological illness and trauma specifically, lack of service provider knowledge about psychological trauma, and health system issues such as interpreter availability. These barriers reflect those found in previous literature [16, 49]. While some aspects of these pathways (e.g., stigma) are relevant for mental health more broadly, this review also identified some specifically trauma related issues—most notably somatisation. The findings of some papers [26, 30, 36, 37] included in this review identified somatisation amongst refugees, which may increase primary healthcare use rather than specifically mental healthcare.

### Recommendations

The studies included in this review made several recommendations to improve healthcare access and utilisation by refugees and asylum seekers. In particular, studies specifically identified comprehensive training of primary care physicians in recognising trauma symptoms, particularly somatisation, as a necessary step in improving the mental health of refugee populations and increasing utilisation of mental health services [26, 28–30, 36, 37, 51–53]. This is particularly important given that the overall findings of the review suggest that refugees are more likely to utilise physical rather than mental health providers. This may be explained by both the fact that those with somatic symptoms are more likely to identify physical—rather than mental—health complaints, as well as the structure of healthcare in many resettlement countries where general practitioners are often responsible for initial mental health reviews and act as the gatekeepers to specialised mental health care, that may entail negotiation of complex referral pathways [54]. As such, there is a clear need for primary healthcare providers who are on the front line of service access (such as general practitioners) to have specialised trauma training—particularly in relation to somatisation as a key symptom of psychological trauma for many refugees [27, 28]; also found in previous research [55].

Other key recommendations included taking into account community understandings of mental health and psychological trauma in provision of services [31], including the use of outreach and community mental health services to work in partnership with communities [10, 25, 36]. This has been identified in research with refugee populations more generally [56, 57] but the presence of psychological trauma arguably makes partnerships more important given the complex symptom profiles associated with trauma. The use of interpreters was also raised [28, 32, 37], reflecting previous research in relation to mental health and general health care [10, 50], highlighting the need for both access to interpreters and specialised mental health care training for interpreters themselves—including in relation to trauma informed care. Reducing cost [36] and addressing resettlement challenges such as food security, housing issues, and employment which may act as barriers due to their immediate priority status for people were also discussed [30, 33, 36]. Wright et al. [33] note that trauma exacerbates these challenges, creating double binds for many refugees where they cannot secure housing (for example) due to trauma symptoms, which in turn prevents them from seeking mental health care. Research into social determinants of health support these recommendations given that these issues will also impact health themselves [58].

Culturally appropriate [10, 26, 36, 37] and trauma-informed care [27, 28, 32] were also advocated, with culturally appropriate methods and trauma informed practice a key feature of broader research [6, 40, 47]. In the case of refugees specifically, this included training in the specific needs of this community rather than broad discussions of culture or psychological trauma. For children and young people, child-appropriate methods such as “toolboxes” to work through emotional expression which may be particularly affected by psychological trauma in children and young people were highlighted as important [34], and this could be particularly the case of unaccompanied minors where it is particularly important that healthcare provides a child-friendly pathway to emotional expression and advocacy. Similarly, developing relationships—including building trust and rapport—were identified in some studies as particularly important for children and young people experiencing psychological trauma [29].

### Limitations

This review has several limitations. In particular, our search strategy included only English language articles from electronic databases which presents a source of bias, particularly given the subject area. It is also worth noting that the broad and often unclear use of the term ‘trauma’ in literature describing refugee experiences required us to narrow our definition in order to focus our analysis on those studies with specific trauma measures or mention of psychological trauma. It is possible that in doing so we excluded some studies with relevant findings, and this is particularly true since trauma is often co-morbid with other mental illness and physical health conditions. Relatedly, the large range of measures used for psychological trauma meant that providing a consistent picture of the impact of psychological trauma on healthcare utilisation was challenging—an issue noted in the field more generally [47]. It is also noteworthy that previous research has found cultural differences in the expression of emotional distress, which also leads to differences in PTSD or trauma symptomatology [1, 29, 41–45]. This makes drawing conclusions across the diverse cultural groups included in this study challenging, and future research would usefully consider the specific effect psychological trauma may have on service access for specific cultural or ethnic groups. Finally, our focus on primary healthcare means that this review is unable to comment on whether not accessing appropriate care may lead to higher presentation to hospitals or emergency services, or indeed the consequences of not accessing care more broadly.

## Conclusion

While a large range of previous research has indicated that refugees and asylum seekers face numerous barriers to accessing primary healthcare—including mental healthcare—this review indicates that people experiencing psychological trauma face a range of additional barriers that warrant specific consideration. This is particularly true for somatisation, which is characteristic of the experience of many refugees [55]. The review therefore indicates a range of implications for both general primary health care and mental healthcare specifically, including more streamlined referral processes into mental health services, training in psychological trauma and somatisation for general healthcare providers, and community and outreach services which may assist in reducing stigma and increasing service access. The review also supports previous calls for research into cross-culturally validated therapeutic tools and increasing availability of interpreters. Overall, there is a need for more robust research concerning psychological trauma and access to care for refugees, in order to ensure appropriate health care.

## Data Availability

Data sharing is not applicable to this article as no datasets were generated or analysed during the current study.

## Abbreviations

CIDI-WHO: Composite International Diagnostic Interview-World Health Organisation
HSC: Hopkins Symptom Checklist
HTQ: Harvard Trauma Questionnaire
MMAT: Multi-Method Appraisal Tool
PTSD: Post-Traumatic Stress Disorder
UNHCR: United Nations High Commission for Refugees
